# Whole-Genome Sequence-Based Characterization of Pre-XDR *M. tuberculosis* Clinical Isolates Collected in Kazakhstan

**DOI:** 10.3390/diagnostics13122005

**Published:** 2023-06-08

**Authors:** Asset Daniyarov, Ainur Akhmetova, Saule Rakhimova, Zhannur Abilova, Dauren Yerezhepov, Lyailya Chingissova, Venera Bismilda, Nurlan Takenov, Ainur Akilzhanova, Ulykbek Kairov, Ulan Kozhamkulov

**Affiliations:** 1 Laboratory of Bioinformatics and Systems Biology, Center for Life Sciences, National Laboratory Astana, Nazarbayev University, Astana 010000, Kazakhstan; asset.daniyarov@nu.edu.kz; 2 Laboratory of Genomic and Personalized Medicine, Center for Life Sciences, National Laboratory Astana, Nazarbayev University, Astana 010000, Kazakhstan; 3 Department of General Biology and Genomics, L.N. Gumilyov Eurasian National University, Astana 010000, Kazakhstan; 4 National Scientific Center of Phthisiopulmonology of the Republic of Kazakhstan, Almaty 050000, Kazakhstan

**Keywords:** tuberculosis, next-generation sequencing, bioinformatics analysis, *Mycobacterium tuberculosis*, pre-extensively drug-resistant

## Abstract

Background: Kazakhstan has a high burden of multidrug-resistant tuberculosis in the Central Asian region. This study aimed to perform genomic characterization of *Mycobacterium tuberculosis* strains obtained from Kazakhstani patients with pre-extensively drug-resistant tuberculosis diagnosed in Kazakhstan. Methods: Whole-genome sequencing was performed on 10 pre-extensively drug-resistant *M. tuberculosis* strains from different regions of Kazakhstan. All strains had high-confidence resistance mutations according to the resistance grading system previously established by the World Health Organization. The genome analysis was performed using TB-Profiler, Mykrobe, CASTB, and ResFinder. Results: Valuable information for understanding the genetic diversity of tuberculosis in Kazakhstan can also be obtained from whole-genome sequencing. The results from the Phenotypic Drug Susceptibility Testing (DST) of bacterial strains were found to be consistent with the drug resistance information obtained from genomic data that characterized all isolates as pre-XDR. This information can help in developing targeted prevention and control strategies based on the local epidemiology of tuberculosis. Furthermore, the data obtained from whole-genome sequencing can help in tracing the transmission pathways of tuberculosis and facilitating early detection of outbreaks. Conclusions: The results from whole-genome sequencing of tuberculosis clinical samples in Kazakhstan provide important insights into the drug resistance patterns and genetic diversity of tuberculosis in the country. These results can contribute to the improvement of tuberculosis control and management programs in Kazakhstan.

## 1. Introduction

According to the World Health Organization’s (WHO) data in 2021, tuberculosis (TB) affected approximately 10.6 million people worldwide, with 6.4 million new diagnoses. TB claimed 1.6 million lives in the same year, including 187,000 people with HIV. TB is currently the 13th leading cause of death and the second leading infectious killer after COVID-19, surpassing HIV/AIDS [1].

In Kazakhstan, the Ministry of Health reports that between 2000 to 3000 people contract multidrug-resistant tuberculosis (MDR-TB) each year, with a 7–8% mortality rate. MDR-TB patients comprise 25% of all TB cases in the country, and the primary cause of drug resistance is the irregular intake of anti-tuberculosis drugs. Despite these challenges, the effectiveness of TB treatment in Kazakhstan was 79.5% at the end of 2018, one of the highest in the world. The number of MDR-TB patients has decreased by 30% over five years, from 10,455 in 2013 to 7282 in 2018. Over the last decade, TB incidence in Kazakhstan has declined by 8–10% annually, with a 24.6% decrease in 2020 compared to 2019. Effective diagnosis and treatment have saved over 53,000 people from TB over the last three years.

In January 2021, the WHO introduced new definitions for extensively drug-resistant tuberculosis (XDR-TB) and pre-extensively drug-resistant tuberculosis (pre-XDR-TB). Pre-XDR-TB refers to *M. tuberculosis* strains that meet the MDR-TB definition and are resistant to rifampicin and any fluoroquinolone. XDR-TB is now defined as TB caused by *M. tuberculosis* strains that are resistant to isoniazid, rifampicin, any fluoroquinolone, and either bedaquiline or linezolid, or both. The emergence of pre-XDR-TB strains represents a critical stage in the progression toward XDR-TB strains. In this study, we utilized whole-genome sequencing (WGS) to predict pre-XDR drug resistance in a selected subset of *M. tuberculosis* isolates from Kazakhstan.

## 2. Materials and Methods

### 2.1. Sample Selection and Processing

The pre-XDR-TB strains from hospitals and public health laboratories throughout Kazakhstan were sent to the National Scientific Center of Phthisiopulmonology (NSCP) of the Ministry of Health of the Republic of Kazakhstan for TB confirmation and evaluation of antimicrobial susceptibility. Drug Susceptibility Testing (DST) of *Mtb* was performed by the absolute concentration method on a solid LJ medium according to WHO recommendations [2] and by using the BACTEC-MGIT 960 Mycobacteria Growth Indicator Tube (BD Diagnostic Systems, United States) system according to the instructions of the manufacturer for the first line (isoniazid, rifampicin, streptomycin, ethambutol, and pyrazinamide) and second line (levofloxacin, moxifloxacin, bedaquiline, linezolid, delamanid, clofazimine, amikacin, kanamycin, prothionamide/ethionamide) anti-TB drugs. Colonies from LJ media were heat killed at 80 °C for 45 min to inactivate the bacteria. Genomic DNA was extracted by using the traditional cetyltrimethylammonium bromide (CTAB) procedure [3] from visible colonies and by PureLink™ Genomic DNA Mini Kit (Thermo Fisher Scientific) according to the manufacturer’s instructions.

### 2.2. Ethics Statement

The studies involving human participants were reviewed and approved by the ethical committee of the Center for Life Sciences, National Laboratory Astana, Nazarbayev University (protocol #05-2020, 24 September 2020).

### 2.3. Library Preparation and Whole-Genome Sequencing

Libraries for WGS were prepared from DNA samples with the Nextera XT kits and run on Illumina next-generation sequencing platforms (MiSeq) as instructed by the manufacturer (Illumina, San Diego, CA, USA). Base calling was performed with bcl2fastq2, version 2.20 software was used to demultiplex samples and generate paired-end fastq reads.

### 2.4. Bioinformatics Processing of Whole-Genome Sequencing Data

Quality evaluation of paired-end reads was performed using FastQC v0.11.9 (https://www.bioinformatics.babraham.ac.uk/projects/fastqc/) (1 March 2022). The generated raw sequencing data in FASTQ format were filtered with Trim Galore (https://www.bioinformatics.babraham.ac.uk/projects/trim_galore/) (1 March 2022) using default values and a minimum Phred score of 20 to remove adapters and poor-quality bases. The sequence reads were aligned to reference strain *M. tuberculosis* H37Rv (NC_000962.3) and variants were called using Samtools mpileup v1.2 [4] and Bcftools v1.10.2 [5]. Mutations with low-quality evidence (i.e., possible mixed read alignment) were not included. Variants with low-quality evidence (i.e., possible mixed read alignment) were not included. Raw FASTQ sequencing files were uploaded to TB-Profiler [6], an online tool for determining drug resistance. The pipeline searches for small variants and big deletions associated with drug resistance. By default, it uses Trimmomatic to trim the reads, bwa [4] to align to the reference genome and GATK (open source v4) [7] to call variants. In silico determination of drug resistance of raw FASTQ sequencing files was performed using four programs (TB-Profiler [6], Mykrobe [8], CASTB [9], and ResFinder [10]). CASTB and ResFinder are online programs. TB-Profiler and Mykrobe tools were run in command line mode. MTB lineages and sublineages determination were performed with the MTBseq tool [11].

### 2.5. Phylogeny Construction

Phylogenetic analysis was performed on ten clinical isolates of MTB and nine reference strains using the CSI Phylogeny tool with the FastTree method. The tree was constructed based on the identification of single-nucleotide polymorphisms (SNPs) from completely sequenced whole-genome sequences of these isolates and strains obtained from the NCBI (RUS_B0—Russian Federation, XDR KZN 605—South Africa, PanR1006—South Africa, str. Beijing/NITR203—India, K—South Korea, H37Rv—UK, H37Ra—France, CDC1551—USA, KZN 4207—South Africa).

### 2.6. Resistance Genetic Variants

Resistance-associated genes were analyzed to evaluate phenotypic resistance to rifampicin (*rpoB*, *rpoC*), isoniazid (*katG*, *fabG1*), ethambutol (*embB*, *embA*), streptomycin (*rpsL*), pyrazinamide (*pncA*), ethionamide (*fabG1*, *ethA*), fluoroquinolones (*gyrA*), amikacin (*rrs*), kanamycin (*eis*), para-aminosalicylic acid (*folC*), and other genes (*mshA*, *mmpR5*, *ald*, *gid*, *ddn*, *tlyA*, *alr*, *ddn*, *fbiB*, *tlyA*).

## 3. Results

### 3.1. Diagnosis and Demographic Data of Patients

Ten patients (five females and five males aged 23–54 years) with complex MTB infections were recruited in 2022. Among these patients, seven were diagnosed with infiltrative pulmonary TB with or without bacterial effusion, one with fibrous cavernous pulmonary TB, one with extrapulmonary tuberculosis (skeletal bone and joint TB), and the last patient with long-term sequelae of respiratory and unspecified tuberculosis (Table 1). The diagnosis was based on clinical features and confirmed by TB culture, smear microscopy, or radiological data. MTB isolates were successfully cultured and sequenced.

### 3.2. Features of Genome Sequencing

The sequence reads from 10 samples were successfully mapped to the H37Rv reference genome with an average read depth of 160X and mean genome coverage of 98.95% (Table 2). The table provides information on various characteristics of these isolates, including drug resistance, total bases, GC content, coverage mean, and coverage median.

Mapping of the raw MTB sequence data led to high average genome-wide coverage across the clinical isolates (median: 160-fold; range: 83- to 515-fold). A total of 10 samples were sequenced, and all of them showed good-quality data. WGS was used to determine the genotype of the 10 samples of *M. tuberculosis*. The results of the NGS reading were compared to the reference strain of *M. tuberculosis* H37Rv, which is displayed in Table 2. The results of the data analysis showed that the largest genome of *M. tuberculosis* in the Kazakhstan strain was 4,357,379 base pairs, and the smallest was 4,288,471 base pairs.

### 3.3. Phenotypic Resistance Patterns

The isolates show a high level of antibiotic resistance, with all isolates being resistant to first-line antibiotics rifampicin (RMP), isoniazid (INH), and pyrazinamide (PZA). All ten *M. tuberculosis* isolates showed drug resistance to first-line anti-TB basic drugs INH, RMP and to one of the tested second-line anti-TB drugs fluoroquinolones (FLQ) (Moxifloxacin or Levofloxacin). All isolates were identified as drug-susceptible for Linezolid (Lzd) and Bedaquiline (Bdq). Therefore, all ten isolates regarding the WHO’s new definitions were classified as pre-extensively drug-resistant tuberculosis (pre-XDR-TB). Nine clinical isolates showed drug resistance to first-line anti-TB drugs PZA, while other anti-TB drugs showed resistance to one or more of the *M. tuberculosis* clinical isolates. Additionally, several isolates are resistant to ethambutol (EMB) and streptomycin (SM). The second-line antibiotics levofloxacin (Lfx), moxifloxacin (Mfx), and prothionamide/ethionamide (Pro/Eto) show the highest level of resistance across the sample group. Some isolates are also resistant to kanamycin (Km), capreomycin (Cm), amikacin (Am), and clofazimine (Cfz) (Table 1). The results from the Phenotypic Drug Susceptibility Testing (DST) of bacterial strains were found to be consistent with the drug resistance information obtained from genomic data that characterized all isolates as pre-XDR.

### 3.4. Antimicrobial Resistance and Drug Resistance-Associated Mutations

The resistance mutations of 10 samples were evaluated and compared to the results of Drug Susceptibility Tests for 10 anti-TB drugs. In all 10 isolates with any drug resistance, the genes *rpsL* and *katG* had a missense variant of Lys43Arg and Ser315Thr, respectively, making them the most commonly mutated genes associated with drug resistance (Table 3). The second most frequently mutated gene associated with drug resistance is *embB*, which had six different missense variants and one upstream gene variant and was present in seven isolates with any drug resistance. The majority of drug resistance-associated mutations are missense variants, which could lead to a change in the amino-acid sequence of the encoded protein. There were also several upstream gene variants, frameshift variants, stop-gained variants, and non-coding transcript exon variants. Table 3 includes 10 isolates with any drug resistance, and each isolate is characterized by its drug resistance-associated mutations. Table 3 lists different drugs and their corresponding genes with amino acid changes associated with drug resistance. These drugs include isoniazid, rifampicin, ethambutol, streptomycin, pyrazinamide, ethionamide, fluoroquinolones, amikacin, kanamycin, and para-aminosalicylic acid. Some genes were associated with drug resistance for more than one drug, such as *katG*, *fabG1*, *rpoB*, *rpoC*, and *embB*.

### 3.5. Antibiotic Resistance Detected by: TB-Profiler, Mykrobe, CASTB, and ResFinder

Identification of drug resistance was performed by using four programs (TB-Profiler, CASTB, Mykrobe, and ResFinder) and is shown in Table 4. All bacterial isolates examined in this study were found to be resistant to isoniazid, rifampicin, ethambutol, and streptomycin. Resistance to pyrazinamide varied among the isolates, with some exhibiting complete resistance (R/R/R/R) while others showed partial resistance. Partial resistance to ethionamide was observed in isolates 232, 304, and 1853, whereas all other isolates showed similar resistance patterns. Samples 163 and 1483 displayed full resistance to amikacin and capreomycin, respectively, while the rest of the samples were susceptible. Additionally, samples 163, 232, 304, 1483, and 2325 showed full or partial resistance to kanamycin, while the remaining samples were susceptible. Isolate 163 showed partial resistance to para-aminosalicylic acid, whereas the other isolates were susceptible. The information regarding aminoglycosides is limited, and only isolate 1483 exhibited partial resistance to this antibiotic. All the isolates in this study showed similar resistance patterns to fluoroquinolones, including ofloxacin, moxifloxacin, levofloxacin, and ciprofloxacin.

Genetic variants in multiple genes associated with drug resistance in *M. tuberculosis* were identified by WGS (Table 3). A total of 10 isolates had mutations in genes associated with resistance to INH, including *katG* (*n* = 10) and *fabG1* (*n* = 1). All samples have missense variants in *katG* (Ser315Thr, Arg463Leu), *rpsL* (Lys43Arg), *gyrA* (Glu21Gln, Ser95Thr, Gly668Asp), *mshA* (Ala187Val), *gid* (Glu92Asp) and upstream gene variant in *rpoC* (-339T > C), *embA* (-590C > T), *rpsL* (-165T > C), *rrs* (-187C > T), *ald* (-32T > C) (Table 3).

### 3.6. Comparison of Phenotypic and Genomic Drug Susceptibility Testing Results

The resistance profiles obtained from the phenotypic testing (Section 3.3 and Table 1) and the genetic analysis using TB-Profiler, Mykrobe, CASTB, and ResFinder (Section 3.5 and Table 4) show some differences in the resistance patterns of the isolates.

In both approaches, all isolates were found to be resistant to first-line antibiotics, including RMP and INH, as indicated by the results obtained from the four databases (TB-Profiler, Mykrobe, CASTB, and ResFinder). Moreover, the TB-Profiler database, known for its specialized TB genome analysis, identified resistance to pyrazinamide (PZA) for all 10 isolates. The phenotypic testing identified resistance to fluoroquinolones (Mfx or Lfx) in all isolates, whereas the genetic analysis showed resistance to FLQ in all isolates, specifically highlighting resistance to moxifloxacin (Mfx) in all samples. However, in contrast, the phenotypic analysis indicated that samples 304 and 1483 were found to be susceptible to levofloxacin (Lfx). This difference may be due to variations in the genetic markers used for detecting resistance. Phenotypic testing identified some isolates’ resistance to EMB, SM, Km, Cm, Am, and Cfz. Both the phenotypic and genomic DST methods provided a perfect match in detecting susceptibility to amikacin. The genetic analysis confirmed resistance to these antibiotics in some isolates but did not provide information for others. Phenotypic testing classified all isolates as drug-susceptible to Bdq and Lzd. However, the genetic analysis currently lacks specific information regarding resistance to Bdq and Lzd. Phenotypic testing revealed that samples 304, 711, 1483, and 1748 were resistant to ethionamide (Eto). In contrast, the genetic analysis showed that samples 232,1853 and 304 exhibited resistance to Eto by TB-Profiler database. However, samples 163, 260, 711, 1155, 1483, 1748, and 2325 were found to be susceptible to Eto based on the genetic analysis.

Overall, the phenotypic resistance profile obtained from the phenotypic testing provides more comprehensive information about resistance to specific antibiotics, including second-line drugs and additional antibiotics. However, the genetic analysis using TB-Profiler, Mykrobe, CASTB, and ResFinder provides insights into the genetic variants associated with drug resistance and can complement the results obtained from phenotypic testing. It is important to note that the differences between the two approaches could be due to variations in the methods used, the specific genetic markers analyzed, and the limitations of each approach. Both phenotypic testing and genetic analysis are valuable for understanding the resistance profiles of the isolates, and a combined approach can provide a more complete characterization of drug resistance in *M. tuberculosis*.

### 3.7. SNP Clustering and Distribution in the M. tuberculosis Genomes

We obtained a list of SNPs in the *M. tuberculosis* genomes. The study utilized the MTBseq tool to identify the lineage and outbreak clade of the ten pre-XDR-TB samples under investigation at the time.

All the isolates were classified as belonging to the Beijing genotype, which is known for its high virulence and association with drug resistance. Five isolates were classified as that belonged to the Central Asia Outbreak (CAO) clade, while three isolates were classified as belonging to the Central Asian sublineage, and two were classified as belonging to the European/Russian strain W148 Outbreak (Table 5).

The CAO clade is a distinct branch of the Beijing genotype that was known for its association with multidrug resistance and increased transmissibility. The Central Asian sublineage was a heterogeneous group, but its most virulent component was similarly named the CAO clade [12].

### 3.8. Phylogenetic Analysis of M. tuberculosis Isolates

**Figure 1:**
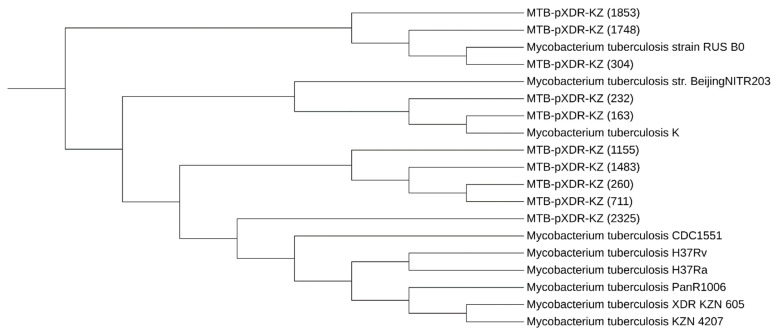
Phylogenetic relationships of *Mycobacterium (M). tuberculosis* isolates based on high-quality SNPs identified by the CSI Phylogeny tool “Reprinted/adapted with permission from Ref. [13] 2011, Rolf S. Kaas” from whole-genome sequences. The tree was constructed by the FastTree method.

Phylogenetic analysis was performed on ten clinical isolates of MTB and nine reference strains (Figure 1).

The resulting tree consists of several major clades, each representing a group of closely related strains. The largest clade, located towards the center of the tree, includes commonly studied strains such as *M. tuberculosis* H37Rv, CDC1551, and H37Ra reference strains.

The tree reveals that strains from Kazakhstan and Russia form a monophyletic group that is genetically distinct from the other strains. Within this group, MTB-pXDR-KZ (1853) and MTB-pXDR-KZ (1748) are closely related, suggesting a recent common ancestor. Additionally, MTB-pXDR-KZ (304) and *M. tuberculosis* strain RUS B0 are also closely related, indicating a possible transmission event.

The remaining strains, including *M. tuberculosis* CDC1551, *M. tuberculosis* PanR1006, and several clinical isolates from different geographic regions, are more distantly related. Notably, *M. tuberculosis* BeijingNITR203 forms a separate branch, indicating it is genetically distinct from all other strains.

The *M. tuberculosis* K strain appears to be closely related to two MTB-pXDR-KZ samples (232, 163), forming a clade with them, suggesting a possible genetic similarity or relationship between the *M. tuberculosis* K strain and these MTB-pXDR-KZ samples.

All of the examined MTB isolates in this study were found to belong to the Beijing lineage (Table 4), which is a highly prevalent and geographically widespread lineage of MTB. The outbreak clade information was available for all the isolates, and the results showed that the majority of the isolates (seven out of ten) were classified as belonging to the Central Asia outbreak clade. Two isolates (304 and 1748) were categorized as part of the European/Russian W148 outbreak clade. One isolate (260) was found to belong to the Beijing lineage but was not associated with any known outbreak clade.

## 4. Discussion

Next-generation sequencing (NGS) nowadays gives us good opportunities for drug resistance detection, and various NGS platforms become more widely available for TB clinical services in many countries. NGS technologies can provide us with complete sequence information for different well-known genes associated with drug resistance for first/second anti-TB drug lines or whole-genomes of clinical isolates of *M. tuberculosis*. Other molecular assays identified a limited set of resistance mutations through the hybridization of probes to specific genetic sequences. The scientific work of many researchers using whole genomic sequencing of *M. tuberculosis* is oriented to studying the TB disease dynamics, the transmission of infection and treatment, the study of small TB outbreaks, and the study of the unique processes of evolutionary dynamics for the spread TB infection [14,15,16].

Previous studies in Kazakhstan were reported about the mutations associated with drug resistance in genes of *M. tuberculosis*. For example, some research work conducted in Kazakhstan was carried out investigating the mutations in genes of *M. tuberculosis* responsible for drug resistance to various anti-TB drugs, especially for rifampicin and isoniazid [17,18,19], molecular genotyping of *M. tuberculosis* [20,21,22,23], and whole-genome sequencing [24,25,26,27]. Hillemann et al. [17] in their study evaluated the possible associations of specific mutations leading to resistance to rifampicin and isoniazid with isolates of the Beijing genotype and isolates of other *M. tuberculosis* genotypes (non-Beijing strains) from Kazakhstan in the early 2000s. In the article by Kozhamkulov et al., mutations associated with drug resistance to rifampicin and isoniazid among Kazakh isolates of *M. tuberculosis* were characterized. A study by Akhmetova et al. [19] described mutations in the *pncA* and *rpsA* genes in pyrazinamide-resistant and pyrazinamide-susceptible *M. tuberculosis* isolates in Kazakhstan, and 12MIRU-VNTR typing was performed to assess the potential use of genotyping in the determination of pyrazinamide resistance. In the work of Ibrayeva et al. [21], the genetic diversity of *M. tuberculosis* isolates distributed in the penitentiary system of Kazakhstan and the civil sector was studied. In the works of Kubica et al. [17] and Skiba et al. [23], genotyping of clinical isolates of *M. tuberculosis* collected in Kazakhstan in 2001 and 2008 was carried out by spoligotyping and IS6110 RFLP methods and using 24 MIRU-VNTR methods and spoligotyping, respectively. Klotoe et al. [22] characterized the genetic diversity of Kazakhstani isolates of *M. tuberculosis* using high-throughput hybridization methods, TB-SPRINT and TB-SNPID typing. According to genotyping data, the Beijing genotype was the predominant genotype in all Kazakhstani studies [19,20,21,22,23].

Only some publications relate to preliminary data and reported several genome sequences of DR (including one XDR and several MDR) clinical isolates of *M. tuberculosis* isolated in Kazakhstan performed by using a next-generation sequencing platform [24,25,26,27,28,29]. Drafts of complete genome sequences of two Kazakhstani isolates [24] and one isolate with extensive drug resistance [26,27] were published by our group. Drug resistance mutations in the genome were reported as a part of the study of the mechanisms of Reversion of Antibiotic Resistance in MDR *Mycobacterium tuberculosis* induced by a Nanomolecular Iodine-Containing Complex FS-1 [25]. The genomes of three drug-resistant clinical isolates of the Latin American-Mediterranean (LAM) family collected in Kazakhstan were reported by Tarlykov et al. [28]. A last study of ours concentrated on WGS MDR-TB isolates from Kazakhstan [29] and identified Lineage 2 East-Asia (Beijing) as a major contributor to the genetic diversity of MTB in the region.

Therefore, we conducted the first WGS study in the country that reports the application of WGS to clinical pre-XDR-TB *M. tuberculosis* isolates from Kazakhstan, demonstrating its utility in managing and controlling tuberculosis in the country. This study illustrated the potential for the identification of drug resistance by using four available analyzing programs (TB-Profiler, CASTB, Mykrobe, and ResFinder) as a method for rapidly diagnosing drug-resistant.

In this study, we investigated the drug resistance patterns of *M. tuberculosis* clinical isolates and found that the concordance rate of the phenotypic DST and WGS DR data were similar for isoniazid, rifampicin, amikacin, fluoroquinolones (Mfx, Lfx) among all 10 isolates. It should be noted similarities of both methods for the identification of MDR, pre-XDR clinical isolates in our study. Resistance to other drugs varied among the isolates, with some showing complete resistance and others showing partial resistance, emphasizing the importance of performing Drug Susceptibility Testing and additional genomic analysis. Some mismatches between phenotypic DST and WGS could be explained by the involvement of other genes in the development of any drug resistance, lack of some mutations in databases, false results of phenotypic DST, laboratory cross-contaminations, etc.

Importantly, para-aminosalicylic acid exhibited efficacy against all isolates except one, suggesting that it could be a promising treatment option. Our results emphasize the urgent need for developing new drugs to combat drug-resistant tuberculosis and highlight the importance of continued monitoring of drug-resistance patterns in clinical isolates. The NGS is a good instrument for finding rare and novel mutations and possible new target genes. These findings are significant as they provide insight into the distribution of MTB genotypes in Kazakhstan and suggest that the Central Asia outbreak clade may be responsible for the high prevalence of pre-XDR-TB and XDR-TB in the region. Furthermore, the identification of specific MTB genotypes can inform the development of effective treatment and control strategies to combat the spread of drug-resistant TB in Kazakhstan and beyond.

## 5. Conclusions

The WHO emphasizes the ability of whole-genome sequencing technologies to generate a complete picture of the drug resistance profile of a clinical sample and the flexibility of sequencing applications to accommodate a growing knowledge base surrounding the association between specific mutations and phenotypic drug resistance *M. tuberculosis*, sequencing-based diagnostics for TB drug resistance present an attractive option for the surveillance of TB drug resistance in various settings [30]. The results presented in this study highlight the importance of genomic analysis in understanding the epidemiology of tuberculosis and emphasize the need for continued surveillance and monitoring of TB outbreaks, particularly in high-risk areas like Central Asia. The whole-genome sequencing illustrated good capability for the detection of mutations in genes associated with drug resistance. In Kazakhstan already implemented representative methods of Line probe assays, the Xpert MTB/RIF, and Xpert Ultra assays (Cepheid, Sunnyvale, CA, USA) for rapid DR detection. We have started the process of implementing NGS for DR detection, and further studies are needed to understand the impact of using WGS *M. tuberculosis* on drug resistance TB diagnostics.

## Figures and Tables

**Table 1:** Diagnosis, demographic data of patients and Phenotypic Drug Susceptibility Testing results.

Isolate	DST Result (Phenotypic)	Diagnosis	Gender	Age	Material	First-Line Antibiotics (RMP, INH, SM, EMB, PZA)	Second-Line Antibiotics (Lfx, Mfx, Lzd, Bdq, Cfz, Km, Cm, Am, Pro/Eto)
MTB-pXDR-KZ (163)	Pre-XDR-TB	Infiltrative pulmonary TB, smear positive	Female	35	Sputum	RMP-resistant, INH-resistant, EMB-resistant, PZA-resistant	Lfx-resistant, Mfx-resistant (antibiotic concentration 0.25 µg/mL), Cfz-resistant, Km-resistant, Cm-resistant, Am-resistant
MTB-pXDR-KZ (232)	Pre-XDR-TB	Infiltrative pulmonary TB, smear negative	Male	29	Sputum	RMP-resistant, INH-resistant, PZA-resistant	Lfx-resistant, Mfx-resistant, Km-resistant
MTB-pXDR-KZ (260)	Pre-XDR-TB	Infiltrative pulmonary TB, smear negative	Female	53	Sputum	RMP-resistant, INH-resistant, PZA-resistant	Lfx-resistant, Mfx-resistant (antibiotic concentration 0.25 µg/mL), Mfx-resistant (antibiotic concentration 1.0 µg/mL)
MTB-pXDR-KZ (304)	Pre-XDR-TB	Long-term sequelae of tuberculosis of the respiratory organs and unspecified tuberculosis, smear negative	Male	38	Sputum	RMP-resistant, INH-resistant, SM-resistant, PZA-resistant	Mfx-resistant, Pro/Eto-resistant
MTB-pXDR-KZ (711)	Pre-XDR-TB	Infiltrative pulmonary TB, smear positive	Female	27	Sputum	RMP-resistant, INH-resistant	Lfx-resistant, Mfx-resistant (antibiotic concentration 0.25 µg/mL), Cm-resistant, Pro/Eto-resistant
MTB-pXDR-KZ (1155)	Pre-XDR-TB	Fibrocystic cavernous TB, smear negative	Male	47	Sputum	RMP-resistant, INH-resistant, EMB-resistant, PZA-resistant	Lfx-resistant, Mfx-resistant (antibiotic concentration 0.25 µg/mL)
MTB-pXDR-KZ (1483)	Pre-XDR-TB	Infiltrative pulmonary TB, smear positive	Female	23	Sputum	RMP-resistant, INH-resistant, EMB-resistant, PZA-resistant	Mfx-resistant (antibiotic concentration 0.25 µg/mL), Km-resistant, Cm-resistant, Pro/Eto-resistant, Am-resistant
MTB-pXDR-KZ (1748)	Pre-XDR-TB	Infiltrative pulmonary TB, smear positive	Male	34	Sputum	RMP-resistant, INH-resistant, SM-resistant, PZA-resistant	Lfx-resistant, Mfx-resistant (antibiotic concentration 0.25 µg/mL), Pro/Eto-resistant
MTB-pXDR-KZ (1853)	Pre-XDR-TB	Infiltrative pulmonary TB, smear positive	Male	53	Sputum	RMP-resistant, INH-resistant, SM-resistant, PZA-resistant	Lfx-resistant, Mfx-resistant (antibiotic concentration 0.25 µg/mL), Cfz-resistant
MTB-pXDR-KZ (2325)	Pre-XDR-TB	Bone and joint TB	Female	40	Pus	RMP-resistant, INH-resistant, EMB-resistant, PZA-resistant	Lfx-resistant, Mfx-resistant (antibiotic concentration 0.25 µg/mL)

Abbreviations: DST—Drug Susceptibility Testing, RMP—Rifampicin, INH—Isoniazid, SM—Streptomycin, EMB—Ethambutol, PZA—Pyrazinamide, Lfx—Levofloxacin, Mfx—Moxifloxacin, Lzd—Linezolid, Bdq—Bedaquiline, Cfz—Clofazimine, Km—Kanamycin, Cm—Capreomycin, Am—Amikacin, Pro/Eto—Prothionamide/Ethionamide.

**Table 2:** Isolate characteristics: data derived from WGS including mapping indicators.

Isolate	Drug-Resistance	Total Bases ^a^	% Total Bases ^a^	GC-Content ^a^	Coverage Mean ^a^	Coverage Median ^a^	Total Bases ^b^	% Total Bases ^b^	GC-Content ^b^	Coverage Mean ^b^	Coverage Median ^b^
MTB-pXDR-KZ (163)	Pre-XDR-TB	4,371,158	0.99	65.59	97.18	97	4,311,226	0.98	65.45	98.34	97
MTB-pXDR-KZ (232)	Pre-XDR-TB	4,355,569	0.99	65.58	170.18	173	4,289,943	0.97	65.41	172.60	174
MTB-pXDR-KZ (260)	Pre-XDR-TB	4,367,295	0.99	65.56	115.97	117	4,289,832	0.97	65.37	117.82	118
MTB-pXDR-KZ (304)	Pre-XDR-TB	4,371,809	0.99	65.58	197.59	199	4,309,013	0.98	65.40	200.25	200
MTB-pXDR-KZ (711)	Pre-XDR-TB	4,376,887	0.99	65.60	150.03	149	4,351,585	0.99	65.51	150.85	150
MTB-pXDR-KZ (1155)	Pre-XDR-TB	4,362,151	0.99	65.56	83.39	83	4,288,471	0.97	65.37	84.64	84
MTB-pXDR-KZ (1483)	Pre-XDR-TB	4,367,543	0.99	65.57	86.92	86	4,307,297	0.98	65.39	88.01	87
MTB-pXDR-KZ (1748)	Pre-XDR-TB	4,374,730	0.99	65.59	99.27	102	4,318,264	0.98	65.44	100.43	102
MTB-pXDR-KZ (1853)	Pre-XDR-TB	4,366,656	0.99	65.58	93.31	95	4,302,788	0.98	65.41	94.54	96
MTB-pXDR-KZ (2325)	Pre-XDR-TB	4,376,075	0.99	65.61	500.20	515	4,357,379	0.99	65.55	502.23	515

^a^ Computed against the respective *M. tuberculosis* reference strain H37Rv (NC_000962.3; 4,411,532 bp) and covered by reads. ^b^ Computed against the respective *M. tuberculosis* reference strain H37Rv and covered unambiguously.

**Table 3:** Distribution of drug resistance-associated mutations in 10 *Mycobacterium tuberculosis* isolates with any drug resistance identified by whole-genome sequencing.

Drug	Gene	Amino Acid Change/Nucleotide Change (Drug Resistance-Associated Mutations)	Type	No. of Isolates	Amino Acid Change/Nucleotide Change (Non-Synonymous Mutations Which Have Not Been Associated with Drug Resistance)	Type	No. of Isolates
Isoniazid	*katG* *fabG1*	p.Ser315Thr (c.944G > C) c.-8T > C	missense variant upstream gene variant	10 1	p.Arg463Leu (c.1388G > T)	missense variant	10
Rifampicin	*rpoB*	p.His445Tyr (c.1333C > T) p.His445Asn (c.1333C > A) p.Ser450Leu (c.1349C > T) p.Leu430Pro (c.1289T > C)	missense variant missense variant missense variant missense variant	1 1 8 1	p.Ile1035Val (c.3103A > G) p.Arg552His (c.1655G > A)	missense variant missense variant	1 1
	*rpoC*	p.Ile491Thr (c.1472T > C)	missense variant	1	c.-339T > C p.Val483Ala (c.1448T > C) p.Glu1092Asp (c.3276A > C) p.Gln435His (c.1305G > C) p.Val483Gly (c.1448T > G) p.Ile491Val (c.1471A > G) p.Asp943Gly (c.2828A > G)	upstream gene variant missense variant missense variant missense variant missense variant missense variant missense variant	10 2 8 1 1 1 1
Ethambutol	*embB*	p.Met306Ile (c.918G > A) p.Met306Val (c.916A > G) p.Gly406Ala (c.1217G > C) c.-12C > T p.Asp354Ala (c.1061A > C) p.Asp1024Asn (c.3070G > A)	missense variant missense variant missense variant upstream gene variant missense variant missense variant	1 3 1 3 2 2			
	*embA*				c.-590C > T	upstream gene variant	10
Streptomycin	*rpsL*	p.Lys43Arg (c.128A > G)	missense variant	10	c.-165T > C	upstream gene variant	10
Pyrazinamide	*pncA*	p.Val7Gly (c.20T > G) c.-724_ * 14839del p.Val139Ala (c.416T > C) c.11A > G p.Ile6Thr (c.17T > C) p.Gly162Asp (c.485G > A) p.Leu182Ser (c.545T > C) p.Trp68Gly (c.202T > G) p.Thr142Met (c.425C > T)	missense variant transcript ablation missense variant missense variant missense variant missense variant missense variant missense variant	1 1 1 1 1 1 1 1 1			
Ethionamide	*fabG1*	c.-8T > C p.Tyr92 * (c.276T > G)	upstream gene variant stop gained	1 1			
	*ethA*	p.Lys37fs (c.110delA) p.Thr61Met (c.182C > T)	frameshift variant missense variant	1 1	p.Leu244Pro (c.731T > C) p.Gln24Pro (c.71A > C) p.Thr314Ile (c.941C > T) p.Arg292 * (c.874C > T)	missense variant missense variant missense variant stop gained	1 1 1 1
Fluoroquinolones	*gyrA*	p.Asp94Gly (c.281A > G) p.Ala90Val (c.269C > T) p.Asp94Asn (c.280G > A)	missense variant missense variant missense variant	4 4 2	p.Glu21Gln (c.61G > C) p.Ser95Thr (c.284G > C) p.Gly668Asp (c.2003G > A)	missense variant missense variant missense variant	10 10 10
Amikacin	*rrs*	n.1401A > G	non-coding transcript exon variant	2	c.-187C > T	upstream gene variant	10
Kanamycin	*eis*	c.-37G > T c.-10G > A c.-8C > A	upstream gene variant upstream gene variant upstream gene variant	1 1 1			
Para-aminosalicylic acid	*folC*	p.Ser150Gly (c.448A > G)	missense variant	1			
	*mshA*				p.Ala187Val (c.560C > T)	missense variant	10
	*mmpR5*				p.Val7fs (c.19delG) p.Cys46Arg (c.136T > C)	frameshift variant missense variant	1 1
	*ald*				c.-32T > C c.-89A > G c.897dupG	upstream gene variant upstream gene variant frameshift variant	10 2 1
	*gid*				p.Glu92Asp (c.276A > C)	missense variant	10
	*ddn*				p.Arg30Ser (c.88C > A)	missense variant	1
	*tlyA*				p.Lys182Thr (c.545A > C)	missense variant	1
	*alr*				p.Lys157Glu (c.469A > G)	missense variant	1
	*ddn*				p.Arg30Ser (c.88C > A)	missense variant	2
	*fbiB*				p.Ser54Ala (c.160T > G)	missense variant	1
	*tlyA*				p.Pro253Ala (c.757C > G)	missense variant	1

* The mutation c.-724_14839del represents a deletion of nucleotides from position c.-724 to an unspecified position, resulting in a frameshift mutation, while the mutations p.Tyr92 and p.Arg292* indicate premature stop codons at positions 92 and 292, respectively, leading to truncated protein products.

**Table 4:** Comparison of Antibiotic Resistance Patterns for TB Isolates Detected by: TB-Profiler, Mykrobe, CASTB, and ResFinder.

Drug	Gene	TB-Profiler/Mykrobe/CASTB/ResFinder									
		**MTB-pXDR-KZ (** **163)**	**MTB-pXDR-KZ (** **232)**	**MTB-pXDR-KZ (260)**	**MTB-pXDR-KZ (304)**	**MTB-pXDR-KZ (711)**	**MTB-pXDR-KZ (1155)**	**MTB-pXDR-KZ (1483)**	**MTB-pXDR-KZ (1748)**	**MTB-pXDR-KZ (1853)**	**MTB-pXDR-KZ (2325)**
Isoniazid	*katG*	R/R/R/R	R/R/R/R	R/R/R/R	R/R/R/R	R/R/R/R	R/R/R/R	R/R/R/R	R/R/R/R	R/R/R/R	R/R/R/R
Rifampicin	*rpoB*	R/R/R/R	R/R/R/R	R/R/R/R	R/R/-/R	R/R/R/R	R/R/R/R	R/R/R/R	R/R/R/R	R/R/R/R	R/R/R/R
Ethambutol	*embB*	R/R/R/R	R/R/R/R	R/R/R/R	R/R/R/R	R/R/R/R	R/R/R/R	R/R/R/R	R/S/-/R	R/R/R/R	R/R/R/R
Streptomycin	*rpsL*	R/R/R/R	R/R/R/R	R/R/R/R	R/R/R/R	R/R/R/R	R/R/R/R	R/R/R/R	R/R/R/R	R/R/R/R	R/R/R/R
Pyrazinamide	*pncA*	R/R/-/R	R/S/-/S	R/R/-/R	R/R/R/R	-/S/-/S	R/R/-/S	R/R/R/R	R/R/R/R	R/R/-/R	R/S/-/R
Ethionamide	*fabG1*	-/-/-/S	R/-/-/S	-/-/-/S	R/-/-/S	-/-/-/S	-/-/-/S	-/-/-/S	-/-/-/S	R/-/-/R	-/-/-/S
Fluoroquinolones	*gyrA*	R/-/-/-	R/-/-/-	R/-/-/-	R/-/-/-	R/-/-/-	R/-/-/-	R/-/-/-	R/-/-/-	R/-/-/-	R/-/-/-
Ofloxacin		R/R/R/-	R/R/R/-	R/R/R/-	R/R/R/-	R/R/R/-	R/R/R/-	R/R/R/-	R/R/R/-	R/R/R/-	R/R/R/-
Moxifloxacin		R/R/-/-	R/R/-/-	R/R/-/-	R/R/-/-	R/R/-/-	R/R/-/-	R/R/-/-	R/R/-/-	R/R/-/-	R/R/-/-
Levofloxacin		R/-/-/-	R/-/-/-	R/-/-/-	R/-/-/-	R/-/-/-	R/-/-/-	R/-/-/-	R/-/-/-	R/-/-/-	R/-/-/-
Ciprofloxacin		R/R/-/S	R/R/-/S	R/R/-/S	R/R/-/S	R/R/-/S	R/R/-/S	R/R/-/S	R/R/-/S	R/R/-/-	R/R/-/S
Amikacin	*rrs*	R/R/R/R	-/S/-/S	-/S/-/S	-/S/-/S	-/S/-/S	-/S/-/S	R/R/R/R	-/S/-/S	-/S/-/S	-/S/-/S
Capreomycin	*rrs*	R/R/-/R	-/S/-/S	-/S/-/S	-/S/-/S	-/S/-/S	-/S/-/S	R/R/-/R	-/S/-/S	-/S/-/S	-/S/-/S
Kanamycin	*rrs*	R/R/-/R	R/S/-/R	-/S/-/S	R/R/-/R	-/S/-/S	-/S/-/S	R/R/-/R	-/S/-/S	-/S/-/S	R/S/-/R
Para-aminosalicylic acid	*folC*	R/-/-/R	-/-/-/S	-/-/-/S	-/-/-/S	-/-/-/S	-/-/-/S	-/-/-/S	-/-/-/S	-/-/-/S	-/-/-/S
Aminoglycosides								R/-/-/-			

**Table 5:** Lineage distribution obtained using MTBseq tool.

Isolate	Lineage	Outbreak Clade
MTB-pXDR-KZ (163)	Beijing	Central Asia outbreak
MTB-pXDR-KZ (232)	Beijing	Central Asia outbreak
MTB-pXDR-KZ (260)	Beijing	Central Asia
MTB-pXDR-KZ (304)	Beijing	European/Russian W148 Outbreak
MTB-pXDR-KZ (711)	Beijing	Central Asia
MTB-pXDR-KZ (1155)	Beijing	Central Asia outbreak
MTB-pXDR-KZ (1483)	Beijing	Central Asia
MTB-pXDR-KZ (1748)	Beijing	European/Russian W148 Outbreak
MTB-pXDR-KZ (1853)	Beijing	Central Asia outbreak
MTB-pXDR-KZ (2325)	Beijing	Central Asia outbreak

## Data Availability

The sequencing data supporting the results of this article are available at the National Center for Biotechnology Information Sequence Read Archive with the following accession number: PRJNA481625.
